# Refining cryo-EM maps using classical and quantum-mechanical density calculations

**DOI:** 10.1063/4.0001058

**Published:** 2025-10-27

**Authors:** Giulia Palermo

**Affiliations:** University of California, Riverside, CA, USA

## Abstract

We present a novel approach to refining cryo-electron microscopy (cryo-EM) maps using quantum mechanical (QM) density calculations. Our method integrates QM-derived electronic densities into cryo-EM maps through a grid- based fitting procedure. This QM-based refinement was applied to the type III-Dv CRISPR system, a powerful effector complex that targets RNA, revealing the coordination of metal ions at the active site. Our approach identified critical interactions involving Mg2+ ions, which are essential for catalytic activity across the three distinct RNA cleavage sites. These findings offer a detailed understanding of the catalytic mechanism, particularly the role of metal ions in stabilizing the scissile phosphate and promoting nucleophilic attack. This work demonstrates the potential of quantum mechanical methods in cryo-EM map refinement, which are not limited to metal binding but can be extended to ligand/drug binding and to the refinement of poorly solved regions.

